# Conceptualising Animal Abuse with an Antisocial Behaviour Framework

**DOI:** 10.3390/ani1010144

**Published:** 2011-01-26

**Authors:** Eleonora Gullone

**Affiliations:** School of Psychology and Psychiatry, Monash University, Monash, Victoria 3800, Australia; E-Mail: Eleonora.gullone@monash.edu; Tel.: +61-3-9905-5374; Fax: +61-3-9905-3948

**Keywords:** animal abuse, antisocial behavior, violence, aggression, comorbidity

## Abstract

**Simple Summary:**

There is increasing acceptance of the links between animal abuse and aggressive or antisocial behaviours toward humans. Nevertheless, researchers and other professionals continue to call for methodologically sound empirical research amongst claims that current animal abuse research is methodologically limited. Below, I argue that current conceptualizations of antisocial and aggressive human behavior logically incorporate animal abuse. Given that the body of empirical evidence available to support of theories of antisocial and aggressive behaviour is large and sound, conceptualization of animal abuse as an aggressive behaviour rather than a behaviour that is somehow different, enables us to confidently promote putting current understanding into practice.

**Abstract:**

This paper reviews current findings in the human aggression and antisocial behaviour literature and those in the animal abuse literature with the aim of highlighting the overlap in conceptualisation. The major aim of this review is to highlight that the co-occurrence between animal abuse behaviours and aggression and violence toward humans can be logically understood through examination of the research evidence for antisocial and aggressive behaviour. From examination through this framework, it is not at all surprising that the two co-occur. Indeed, it would be surprising if they did not. Animal abuse is one expression of antisocial behaviour. What is also known from the extensive antisocial behaviour literature is that antisocial behaviours co-occur such that the presence of one form of antisocial behaviour is highly predictive of the presence of other antisocial behaviours. From such a framework, it becomes evident that animal abuse should be considered an important indicator of antisocial behaviour and violence as are other aggressive and antisocial behaviours. The implications of such a stance are that law enforcement, health and other professionals should not minimize the presence of animal abuse in their law enforcement, prevention, and treatment decisions.

## 1. Introduction

Antisocial behaviours including aggression and violence, are disruptive, not only to the individual but also to society and the community [1]. A history of antisocial behaviour is predictive of a large range of problems during adulthood including criminal behaviour, work failure, and troubled marriages. At the more extreme end of the antisocial behaviours continuum is violence which has been reported to be one of the leading public health problems worldwide with over 1.6 million lives lost each year and countless more being harmed [2].

Over the past decade, it has also become increasingly clear that aggressive behaviours mostly occur within the context of other antisocial behaviours including lying, stealing, destruction of property, burglary, sexual assault and other violent crimes [3]. Given the co-occurrence of aggressive behaviour, most notably physical aggression with other forms of antisocial behaviour, the focus of research has broadened from the traditional focus on aggressive behaviour to one in which aggression is viewed within the broader framework of antisocial behaviours [4]. Pertinently, as stated by Dishion and colleagues [1], “the frequency and variety of antisocial acts are the best predictors of more serious forms of antisocial behaviour, including violence.” (p. 422).

## 2. Definitions

### 2.1. Antisocial Behaviour and Aggression

Loeber [5] has defined antisocial behaviour as that which causes mental or physical harm, property loss, or damage to others. Frick and Viding [6] provide a somewhat more refined definition of antisocial behaviour as “criminal and aggressive behaviors that violate the rights of others or major societal norms.” (p. 1111).

Human aggression has been defined as behaviour performed by a person (the aggressor) with the deliberate intention of harming another person (the victim) who is believed by the aggressor to be motivated to avoid that harm. Within this context, “harm” refers both to physical (e.g., Punching someone) and psychological (e.g., Verbal abuse) harm. It is also noteworthy that violence is conceptualized as a particularly extreme sub-type of aggression (e.g., murder, rape, assault) [7].

In relation to the multidimensional nature of aggressive behaviour, it can be considered along dimensions including (i) the degree of hostile *versus* agitated affect that is present; (ii) the underlying motive along the dimension of the degree to which the primary or ultimate goal is to cause harm to the victim *versus* the instrumental goal of the perpetrator deriving a profit or reward through the aggressive behaviour, and (iii) the degree to which the likely consequences were considered, reflecting whether the aggressive behaviour was premeditated (thoughtful, deliberate, slow and instrumental) or impulsive (automatic, fast, affect laden). Anderson and Huesmann [8] have also stated that regardless of where the aggressive behaviour falls on the above dimensions, the intention to harm is still a *necessary* goal. This last point is important within the present context since if we are to draw useful conceptual parallels between human aggression and harm perpetrated against animals, given the broad-ranging utilitarian attitudes toward non-human animals (e.g., farming and husbandry practices), conceptualising behaviours serving a purely instrumental goal in the absence of intention to harm as examples of aggression would be problematic.

### 2.2. Animal Abuse

Ascione [9] has defined animal abuse as “socially unacceptable behaviour that intentionally causes unnecessary pain, suffering, or distress to and/or the death of an animal” (p. 51). Consistent with human aggression definitions, this definition of animal abuse and most others (e.g., “the wilful infliction of harm, injury, and intended pain on a nonhuman animal”; p. 1114) [10] include acts of abuse that are *intended* to cause either physical or psychological suffering.

Following a detailed consideration of a number of definitions of animal abuse, including Ascione's [9], Dadds, Turner, and McAloon [11] have noted that most definitions comprise a behavioural dimension including both acts of omission (e.g., neglect) and acts of commission (e.g., beating). Thus an important dimension of animal abuse is indication that the behaviour occurred purposely, that is, with deliberateness and without ignorance. The requirement of *deliberate intention to cause harm* excludes behaviours that cause pain, suffering or distress to animals as a consequence of other behaviours such as, for example, veterinary procedures or practices that are part of animal husbandry (e.g., tail docking without an anaesthetic in factory farmed pigs) and general farming practices even though the end result is the killing of animals, often with measurable suffering involved.

Thus, animal abuse can be defined as behaviour performed by an individual with the deliberate intention of causing harm (*i.e.*, pain, suffering, distress and/or death) to an animal with the understanding that the animal is motivated to avoid that harm. Included in this definition are both physical harm and psychological harm. As per the literature on human aggression, animal abuse at the more extreme end of the aggression dimension (e.g., burning whilst alive, torture—*c.f.*, murder, rape, assault *versus*, for example, teasing, hitting, tormenting), should be considered to be a violent sub-type of animal abuse, and consequently one that should be of particular concern to officials and legislators. Indeed, more consideration needs to be given to the severity of acts of animal abuse than is currently the case. In this regard, considering the classification of the underlying motivations of animal abuse [10] is likely to be most useful.

### 2.3. Animal Abuse Motivations

A number of authors [11,12,13] have emphasised the importance of determining the motivations underlying animal abuse in order to better understand the behaviour, and particularly its relationship with human violence and aggression. To this end, Kellert and Felthous [10] proposed nine categories of motivations including (i) attempts to control an animal (e.g., hitting a dog to stop it barking), (ii) retaliation (e.g., use of extreme punishment for a perceived transgression on the part of the animal such as throwing a cat against a wall for vomiting in the house), (iii) acting out of prejudice against a particular species or breed. Such a motivation is accompanied by the belief that the particular animal is not worthy of moral consideration, (iv) the expression of aggression through an animal (e.g., organising dog fights), (v) acting out of the motivation to enhance one's own aggression (e.g., using animals for target practice or to impress others), (vi) to shock people for amusement (abuse that is very overt and observed by others), (vii) to retaliate against another person or as revenge (e.g., killing or maiming the companion animal of a disliked neighbour), (viii) displacement of aggression from a person to an animal, and (xi) non-specific sadism which refers to the desire to inflict suffering, injury or death in the absence of any particular or hostile feelings toward an animal. A primary goal expressed within this motivation was to derive pleasure from causing the suffering. This motive was explained by Kellert and Felthous as sometimes being related to a desire to exercise power and control over an animal as a way of compensating for feelings of weakness or vulnerability.

Relating to the displacement of aggression (viii), frustrated aggression is typically involved. Many of the aggressive participants in Kellert and Felthous' study reported being physically abused as children. Participants' self-reports were supportive of displaced aggression, typically involving authority figures who they reported hating or fearing. Their abuse of animals reportedly served as a displaced expression of the violence they experienced. As stated by Kellert and Felthous [10], “It is often easier in childhood to be violent toward an animal than against a parent, sibling, or adult.” (p. 1124). Indeed, *displaced aggression* has been described to be a robust phenomenon in the human aggression literature [14,15].

With regard to acting out of prejudice against a particular species or breed (v), this motivation can be best understood with reference to Bandura's [16,17] moral disengagement theory. According to Bandura, certain mechanisms can explain why and when even people who otherwise have normal or even high moral standards sometimes behave in ways that could be considered reprehensible. Two particularly well researched mechanisms are: (i) the cognitive construction of moral justification and (ii) dehumanizing the victim (in the case of animals, this could be conceived of as minimizing the worth/sentience of an animal). Examples of justification include “It is important for the well being of our society.” as could apply to soldiers fighting at war, or “It is for their own good” as could apply after beating a child in the name of discipline, or for *personal honour* as would apply particularly in the case of high self-esteem threats (e.g., beating up the wife to show her who's boss!). Regarding the mechanisms related to dehumanizing the victim, these essentially ensure that the redefinition of the victim is such that personal moral standards no longer apply. Renaming certain animal species as “pests” achieves this aim.

Importantly, Kellert and Felthous [10] noted that despite the ability to list nine separate motivation categories, their data highlighted the multidimensionality of animal abuse where one motivation alone rarely applied. The multidimensionality highlighted by Kellert and Felthous is also a predominant characteristic of aggression toward humans.

The application of the literature regarding human aggression and antisocial behaviour to enhance our understanding of animal abuse may be criticized as not appropriate for a number of reasons, the primary one being that the status occupied by non-human animals in society is different to that applied to humans. This is particularly the case with animals who are classified as “stock” or “produce”, “game” or “vermin”. It is significantly less true, however, for animals afforded “companion” status, many of who are, in today's society, commonly regarded to be members of the family and who are often lavished with the care and nurturance provided to human family members, so much so that legal custody battles in instances of divorce in the family are not unheard of. Indeed, in their review of the relationships between childhood cruelty to animals and later aggression against people, Felthous and Kellert [18] argue that “repeated acts of serious cruelty to socially valued animals (e.g., dogs) are more apt to be associated with violence toward people than are isolated acts of cruelty, minor abuses, and victimization of less socially valuable species (e.g., rats).” (p. 714).

Such an argument is consistent with moral disengagement theory and its relevance is supported by findings reported by Felthous and Kellert [19]. They investigated psychosocial factors in animal abuse based on reports of 23 participants with a history of substantial animal abuse and found that participant reports indicated attitudes toward the animals they had abused as being “worthless objects, hated objects, or narcissistic objects” (p. 1720). Thus, it can be argued that processes of moral disengagement applied in the human aggression literature, are to some degree, normative in relation to non-human animals, albeit less so in relation to companion animals. Despite such differences however, the deliberate infliction of animal suffering is considered to be a criminal behaviour in most countries around the world, reflecting community attitudes that such behaviour is by definition deviant. As such, with regard to behaviours that have the intentional suffering of an animal or animals as a goal, as opposed to some other utilitarian end (e.g., food), it is a reasonable to argue that the application of conceptualisations and understandings documented in the human aggression literature to understandings of animal abuse, is logically defensible.

In addition to understanding animal abusers' motivations for their aggressive behaviour, Hensley and Tallichet [12] point out that understanding perpetrators' characteristics and situational circumstances is of importance. Here too, application of knowledge gained from the human aggression literature is likely to lead to conceptual advances. Such knowledge includes that individual difference variables, environmental experiences, and their interaction, are key to gaining a more comprehensive understanding of the fabric of abuse. Supportive of such a position is the fact that studies into animal abuse have documented similar predictors and developmental pathways of animal abuse when compared to interpersonal antisocial behaviour as well as aggression and violence. The discussion will now turn to this literature.

### 2.4. Developmental Pathways of Antisocial Behaviour

#### 2.4.1. Learning Pathways

Of particular relevance to environmental and psychological aetiological factors of antisocial behaviour and aggression is Bandura's [20] social learning theory of aggression. Proposals regarding acquisition pathways of aggression that have developed from Bandura's theory include those yielded from the work of Patterson, DeBaryshe, and Ramsey [21]. These researchers proposed that maladaptive learning processes can be found in families of aggressive children with central factors including poor parental disciplinary strategies and inadequate monitoring of children's activities. Also of relevance are childrearing characteristics proposed to create bullies [22]. Such characteristics include parental attitudes of indifference toward the child, permissiveness of aggressive behaviour by the child, the use of physical punishment (*i.e.*, the modelling of aggression), and power assertive disciplinary strategies.

#### 2.4.2. Cross-Generational Stability

At more extreme levels are child abuse and child neglect which are now commonly accepted to be factors that place abused or neglected children at increased risk of themselves becoming abusing or neglecting parents [23,24,25,26]. Also, as noted by Repetti, Taylor, and Seeman [27], “Risky families are characterized by conflict, anger, and aggression, by relationships that lack warmth and support, and by neglect of the needs of offspring.” (p. 356). Cross-sectional and prospective research overwhelmingly documents that overt conflict and aggression in the family are associated with increased risk of emotional and behavioural problems in children, including aggression, conduct disorder, delinquency and antisocial behaviour, anxiety, depression, and suicide [27].

#### 2.4.3. Childhood Experiences of Abuse and Engagement in Animal Abuse

In one of the earliest existing studies showing a relationship between animal abuse and human aggression, Tapia [28] reported that among boys with a history of animal abuse, parental abuse was the most common explaining factor. Similarly, in their work comparing criminal (aggressive *versus* non-aggressive) and non-criminal retrospective reports of childhood experiences and abuse behaviours, Kellert and Felthous reported that domestic violence and particularly paternal abuse and alcoholism were factors that were common among aggressive criminals with a history of childhood abuse of animals [10,29,30].

Specifically, Kellert and Felthous [10] reported that the family and childhood experiences of many of the aggressive criminals were particularly violent. The domestic violence in the families of the aggressive criminals was most strongly characterised by paternal violence. Of note, three quarters (75%) of the aggressive criminals reported repeated and excessive child abuse compared to 31% of the non-aggressive criminals and 10% of the non-criminals. Among the non-aggressive criminals and non-criminals who were cruel to animals, reports of being physically abused as children were more common. As many as 75% of non-criminals who reported experiences of parental abuse also reported being cruel to animals.

Whilst the research by Felthous and Kellert can be criticized on the basis of its methodology (*i.e.*, retrospective reports), a more recent study by Duncan, Thomas, and Miller [31] found converging findings through the assessment of charts of boys (aged 8 to 17 years) with conduct problems. Among these children, histories of physical child abuse, sexual child abuse, paternal alcoholism, paternal unavailability, and domestic violence were assessed. Children were grouped according to whether they had been abusive toward animals or not. It was found that children in the abusive group were twice more likely to have been physically and/or sexually abused or to have been exposed to domestic violence compared to the non-abusive group. No differences were found on the other variables assessed, however the findings are limited by the fact that the method of data collection used (*i.e.*, charts) has traditionally been documented as being a gross method of assessment and thus as having compromised reliability. Other group relevant differences may therefore have been missed.

As noted earlier, the abuse of animals has been proposed to constitute, in part the displacement of aggression from humans to animals that occurs through the child's identification with their abuser. By identifying with their abuser, children's sense of powerlessness can be transformed into a sense of control or empowerment. This relates to another of Kellert and Felthous' motivations for abuse wherein a sense of control over an animal is gained through the abuse and may be motivated by a desire to compensate for feelings of weakness or vulnerability. Such an explanation is consistent with Bandura's [20] social learning theory of aggression which argues that caregiver modelling of aggressive behaviours and power assertive disciplinary strategies are predictive of the development of aggressive behaviours. Cognitive theories are also useful in explaining the processes of acquisition of animal abuse behaviours, through the construct referred to as “knowledge structures”. Given that knowledge structures are proposed to develop largely as a consequence of learning experiences, on the basis of theory it would be expected that individuals who experience abuse in their formative years learn specific aggressive behaviours and hostile perceptions, attributions, and expectation biases. They also learn callous attitudes and how to disengage normative empathic reactions that would otherwise serve as aggression inhibitors (and/or otherwise normative development of empathy is suppressed) [32]. Indeed such processes have been implicated in the development of conduct problems and disorders in children [27].

#### 2.4.4. Co-occurrence of Conduct Problem Behaviour and Animal Abuse

Conduct Disorder (CD) has been defined in the DSM-IV as “a repetitive and persistent pattern of behaviour in which the basic rights of others or major age-appropriate societal norms or rules are violated” (p. 98) [33]. The onset of Conduct Disorder (CD) may occur as early as 5 to 6 years of age but more commonly occurs in late childhood or early adolescence. Although as many as 50% of childhood cases of CD remit by adolescence, adolescent cases of CD rarely begin without warning signs in childhood [34].

Reported statistics indicate that CD is one of the most frequently diagnosed childhood conditions, in both outpatient and inpatient mental health facilities, particularly in urban areas. Consistent with the general conceptualisation of externalising behaviours, CD comprises a cluster of oppositional and antisocial behaviours including excessive noncompliance, stealing, lying, running away, physical violence, cruelty (to human and animals), and sexually coercive behaviours [34]. These behaviours are clearly diverse. However, they all share the common characteristic that they violate major social rules and expectations [35]. The disorder is also characterised by constant conflict with others (specifically parents, teachers, and the peer group), and CD in childhood is predictive of other psychological disorders including delinquency, drug abuse, school dropout, suicide and criminality in adolescence or adulthood [34]. Indeed, cases of CD beginning in childhood account for almost half of all adolescent crime [36] and as many as 75% of youth with CD progress to antisocial personality disorder in adulthood [37]. Further supportive of continuity, studies have found that antisocial behaviour in adulthood begins in childhood [37,38].

Diagnostic criteria for CD in the DSM-III [39] and subsequent revised versions include abuse of animals as one criterion. Of particular significance, in their meta-analysis of child conduct problem behaviours, Frick *et al.*, [40] reported a median age of 6.5 years for the occurrence of the first incident of animal abuse along with other aggressive behaviours including fighting (6 years), bullying (median age 7 years), and assaulting (7.5 years). Therefore, animal abuse has been found to be one of the earliest indicators of CD, and is listed as such in the DSM-IV-TR version [33]. Further, as many as 25% of children diagnosed with Conduct Disorder display cruelty to animals. In their analysis of the National Epidemiological Survey data set including a U.S. nationally representative sample of 43,093 respondents, Gelhorn, *et al.*, [37] found that cruelty to animals (assessed with the item “Hurt or be cruel to an animal or pet on purpose?”) significantly discriminated between those with clinical and sub-clinical conduct problem behaviours. Specifically, 5.5% of males in the sub-clinical group compared to 18% of males in the CD group endorsed the item of animal cruelty. The comparative statistics for females were lower but equally discriminating (*i.e.*, 2.2% *versus* 6.2%).

Consistent findings have been reported by Luck, Staiger, Wong and Mathai [41], in their comparison study of 141 clinic-referred children presenting with at least one definite CD symptom apart from animal abuse, and a community sample of 36 children, all aged between 5 and 12 years. Forty children in the clinic-referred group (out of 28–141%) compared to one child from the community sample (3%) were rated as sometimes or definitely being cruel to animals (CTA). The findings revealed a trend for the CTA group to be characterised by poorer family functioning. It was also found that those in the CTA group were reported to have more severe conduct problems and to be more likely to be male. An additional finding was that the older children in the CTA group appeared to have a highly elevated self-perception. Luk and colleagues proposed that the elevated self-worth of their CTA sample, may have be suggestive of the callous and unemotional (CU) trait in children identified in the work by Frick, O'Brien, Wootton, and McBurnett [42]. This trait has been found to manifest as behaviour characterised by lack of guilt and empathy and superficial charm. Of note, such a cluster of characteristics is similar to the concept of psychopathy in adulthood and is consistent with findings related to the precipitants of aggression in the form of threats to an unstable and inflated sense of self [43,44,45].

In addition to being linked with CD, animal abuse has been shown to co-occur with bullying behaviours. Further, both animal abuse and bullying have been related to later antisocial behaviours and antisocial personality disorder [37]. Conceptually, animal abuse and bullying behaviours in youth are analogous. Similarities are apparent in the definitions of animal abuse and bullying. A recent definition of bullying indicates that the behaviour involves a desire to hurt, a power imbalance, an unjust use of power, enjoyment by the aggressor and a general sense of being oppressed on the part of the victim [46]. It is generally agreed that a definition of bullying needs to include an intention to inflict either verbal, physical or psychological harm, a victim who does not provoke the bullying behaviours, and occurrences in familiar social groups [47,48,49,50].

Whilst explicit in definitions of bullying but not in definitions of animal abuse, there is a clear power imbalance where the perpetrator is more powerful than the victim and uses this power to inflict physical, emotional or psychological harm on the victim. Also, both animal abuse and bullying behaviours are predominantly observed in male populations. Research has indicated that males have rates of animal abuse that are four times higher than those of females [51], and that males are more likely than females to engage in bullying behaviours [47,52,53,54]. Further suggesting potentially overlapping processes between animal abuse and bullying is their appearance within a close developmental timeframe. However, despite the strong conceptual overlap, with few exceptions, animal abuse and bullying behaviours have traditionally been researched separately.

#### 2.4.5. Exposure to Aggression and Aggressive Environments

Also demonstrating support for the empirical overlap between animal abuse and human aggression is research examining the witnessing or experiencing of aggression or [55]. Identified problems predicted by such experiences include an increased likelihood of developing beliefs that support aggression [56] and a tendency to behave violently [7].

Supporting the premise that exposure to violence and victimisation experiences are related to animal abuse [12,57], Baldry [58] found that youth who witnessed violence between family members, or who witnessed harm to animals, were three times more likely to have themselved abused animals compared to peers without such experiences.

Baldry's [58] results indicated that girls and boys who had engaged in direct bullying behaviours were twice as likely to have abused animals compared with their non-bullying peers. Engagement in animal abuse by boys was predicted by their direct victimisation at school and indirect bullying, while engagement in animal abuse by girls was predicted by their exposure to animal abuse and by their experience of verbal abuse by their fathers.

In relation to the witnessing of family or domestic violence, Baldry [59] found that children who engaged in bullying behaviours were 1.8 times more likely to have been exposed to domestic violence than those who did not. Exposure to adult aggression and conflict [60] has also been shown to be associated with increased engagement in bullying behaviours.

Gullone and Robertson [61] investigated relationships between self-reported animal abuse and bullying behaviours in a school-based sample of 249 adolescents (105 males, 144 females) ranging in age from 12 to 16 years. Significant positive relationships were found between bullying and animal abuse. Both behaviours were also found to correlate significantly with bullying victimization, witnessing of animal abuse and family conflict. Confirming previous findings regarding sex differences in animal abuse, boys were found to score significantly higher than girls on both animal abuse and bullying.

When examining possible pathways of acquisition between animal abuse and bullying behaviours, it was found that each type of behaviour was significantly predicted by the witnessing of animal abuse. Thus, not only did Gullone and Robertson provide empirical support for the co-existence of animal directed aggression and human directed aggression in youth, as with Baldry's [58] results, they also demonstrated support for the important pathway of observational learning in the development of aggressive behaviour, as predicted by Bandura's [20] social learning theory of aggression.

In a study specifically examining the relationship between the witnessing of animal abuse and engaging in the behaviour, Thompson and Gullone [62] surveyed a total of 281 (113 males; 168 females) school-based adolescents ranging in age between 12 and 18 years. They found that those who reported having witnessed animal abuse on at least one occasion reported significantly higher levels of animal abuse when compared to those youth who reported never having witnessed such abuse. Of particular note is the finding that youth who reported witnessing a stranger abuse an animal, reported significantly *lower* levels of animal abuse. This contrasted with the finding that witnessing of animal abuse by a friend, relative, parent, or sibling related to higher levels of abuse when compared to not witnessing abuse by someone in these categories. These findings support the vicarious learning theory claim that observation of behaviour is more likely to have an impact on the acquisition of the observed behaviour if the model has a meaningful relationship with the observer. An additional important finding was related to the frequency of witnessing abuse such that, as witnessing frequency increased, rates of animal abuse also increased.

While several studies [58,61,62,63] have demonstrated a relationship between witnessing of abuse and engaging in such behaviour via youth self-report, others [12,51,57,64] have demonstrated the relationship by asking undergraduate students or imprisoned males about their childhood experiences and behaviours, albeit through retrospective reports. Currie [65] also reported a significant relationship between the witnessing of aggressive behaviour (domestic violence) and animal abuse via parent-report. Mother reports regarding their children's animal abuse were compared for a group of 94 children (47 mothers) with a history of domestic violence and 90 children (45 mothers) without a history of domestic violence. Exposed children, according to their mothers, were more likely to abuse animals compared to children who were not exposed to violence. All of the above studies point to the witnessing of animal abuse (*i.e.*, an aggressive behaviour) as being an important predictor of the learning of, and engagement in, aggressive behaviour. As noted earlier, children who witness or directly experience violence or aggression have been documented to be more likely to develop beliefs and scripts that support aggression [56] and a tendency to behave aggressively [8]. On the basis of these findings, it can be concluded that animal abuse is a marker of other potentially sinister experiences in children's lives.

The relationship between animal abuse and aggression extends beyond the earlier developmental periods into adulthood. Thus, animal abuse can also be an important indicator of potential aggression toward human adults.

### 2.5. Comorbidity between Human Aggression and Animal Abuse during Adulthood

One of the most consistently replicated findings supporting a link between human violence and animal abuse is that of significant co-occurrence between family or domestic violence and animal abuse. Recent studies have indicated that more than one half of all abused women have companion animals, that many of these companion animals (in as many as 50% of cases) are abused by the perpetrators of the domestic violence as a means of hurting and/or controlling the women or their children, and that concerns for the safety of their companion animals keep many women (and their children) from leaving or staying separated from their abusers.

Ascione [66] has recently reviewed the literature of the relation between animal abuse and the violence experienced by women by their intimate adult partners within the family environment. Several such studies have now been conducted [57,67,68,69,70,71] across several countries including the United States, Canada, and Australia, and the findings have remarkable consistency despite study differences (e.g., country, sample size, methodology). Findings include that between 11.8% and 39.4% of women have reported that the perpetrator *threatened to* hurt or kill their companion animals. Between 25.6% [57] and 79.3% [69] of women reported that the perpetrator had *actually* hurt or killed their companion animal(s).

A limitation of all but two of these studies [70,71] is that they did not include a comparison group of women who were not in a violent family situation. In the Ascione *et al.*, study [71], 5% of non-abused women reported pet abuse and in Volant *et al.*'s study, 0% reported pet abuse [70]. Volant *et al.*'s study involved a group of 102 women recruited through 24 domestic violence services in the state of Victoria and a non-domestic violence comparison group (102 women) recruited from the community. These researchers also found that 46% of women in the domestic violence sample reported that their partner had *threatened to* hurt or kill their pet compared with 6% of women in the community sample [70].

Data have also been obtained relating to the children's witnessing of the animal abuse and the children's abuse of animals. Studies have reported that between 29 and 75% of children in violent families have witnessed the animal abuse and between 10 and 57% of children in these homes have been reported to engage in animal abuse. As noted by Ascione [66], parental reports of animal abuse in normative samples of children are typically around 10% or lower. Not surprisingly, these results are consistent with other studies reporting that children exposed to domestic violence are more likely to engage in acts of animal abuse than children who have not been exposed to domestic violence [58,65,72].

An additional finding derived from studies investigating the co-occurrence of domestic violence and pet abuse is that between 18% [67] and 48% [73] of women have reported delaying leaving their violent situation out of fear that their companion animal(s) would be harmed or killed in their absence.

In addition to domestic violence, research has shown that animal abuse is predictive of other types of criminal behaviours. Arluke, Levin, Luke, and Ascione [74] obtained their data from official records of criminality. Their study also included a comparison group. The researchers identified people who had been prosecuted for at least one form of animal cruelty from the records of the Massachusetts Society for the Prevention of Cruelty to Animals (MSPCA) between 1975 and 1986. They defined animal abuse as cases “where an animal has been intentionally harmed physically (e.g., beaten, stabbed, shot, hanged, drowned, stoned, burned, strangled, driven over, or thrown).” (p. 966). Their sample comprised of 153 participants of whom 146 were male. The comparison group was constituted from individuals matched to the abuse group on variables including gender, socioeconomic status, and age. The study results indicated that animal abusers were significantly more likely than the comparison group participants to be involved in some form of criminal behaviour, including violent offences. Specifically, 70% of those who abused animals also committed at least one other offence compared with 22% of the control group participants. The differences ranged from 11% for the control group and 44% for the abusive group on property-related crimes to 12% for the control group and 37% for the abusive group on public disorder related crimes. For violent crimes, the two groups differed substantially (7% and 37% for the control and abusive groups, respectively). Based on their findings, the authors concluded that a single known act of animal abuse was significantly predictive of increased participation in other criminal offences when compared to a matched sample of adults who did not abuse animals.

Australian Victoria Police data provide support for the findings reported above. Data were obtained from the Statistical Services Division of Victoria Police for all recorded offences in Victoria, Australia for the years 1994 to 2001 (inclusive). Out of four categories of offence (Offences against the person, Offences against property, Drug offences, Other offences), for all alleged offenders, the data clearly showed that the largest proportion of offences was consistently found to be that against property, ranging between 79.52% (number = 344,905) of total offences in 1998 and 80.85% (number = 354,785) in 1999. Over the eight year period, offences against property constituted 80.8% of the total of 3,364,078 crimes committed in Victoria. Drug offences consistently constituted the smallest proportion and ranged between 2.84% (n = 12,838) in 2001 and 4.23% (n = 18,354) of total offences in 1998. Of note, offences against the person also constituted a relatively small proportion of the total number of crimes at an average of 7.71% of all crimes over the eight year period with the lowest percentage of 7.98 recorded in 2000 and the highest percentage of 8.01 recorded in 2001.

The equivalent statistics relating to criminal offences, classified into the same four categories nominated above, but for alleged animal abuse offenders only, revealed that, for animal abuse offenders, the average percentage of offences committed against the person was substantially higher compared to the percentage for all alleged offenders (25% compared to 8%). The category of offences against the person included such crimes as homicide, rape, assault, abduction/kidnap, and harassment. Importantly, these statistics are remarkably similar to those reported by Arluke *et al.*, [74] as described above. Thus, there appears to be a greater likelihood that people alleged to have abused animals will engage in offences against the person, including violent crimes, when compared to all alleged offenders.

Of note, when broken down by age and sex, the data showed that, across crime categories, alleged offenders (all alleged offenders, not only animal abuse offenders) were characteristically male. Also, in general, for the Victorian population, the prevalence of alleged offences during the documented time was highest between the ages of 12 and 35 years for both males and females with a peak between the ages of 18 and 25 years. When examining age and sex trends for alleged animal abuse offenders and animal abuse offences only, the same peak in frequency between the ages of 18 and 25 years was found. Thus, in sum, males were overrepresented for both general alleged offences and alleged animal abuse offences. Males were also overrepresented across all age categories for both general alleged offences and specifically for animal abuse offences, with very few exceptions. Further, a peak of offending was observed between the ages of 18 to 25 years that decreased steadily beyond these years. The particular importance of these statistics is that human aggression and criminal behaviour is demographically parallel along age and sex lines with animal abuse behaviour. This provides additional support for a link between human aggression and animal abuse and strengthens the argument that animal abuse can be most usefully conceptualised within a human aggression framework.

## 3. Conclusions

Studies examining animal abuse have been criticised as having a number of limitations including, problematic methodologies such as retrospective reporting, restricted generalizability of samples (e.g., incarcerated adults), lack of adequate control or comparison groups, and application of different definitions of animal abuse. However, despite these identified limitations, across very different respondent groups (e.g., school-based youth, women from violent homes, incarcerated adults, undergraduate students) and methods (e.g., self-reports, third-party reports, analysis of criminal records), the co-occurrence between human-directed and animal-directed aggression and violence continues to emerge. Evidence is also accumulating to support shared pathways of acquisition of these aggressive behaviours including most significantly the important role played by the direct experiencing of the aggressive or violent behaviour particularly in the form of child abuse, and that played by exposure to, or witnessing of, aggression. There has for sometime now been strong acceptance of these pathways of acquisition for human-directed aggression. Given the clear conceptual overlap of human-directed aggression and animal abuse and given the increasingly strong empirical evidence for the co-occurrence of these behaviours beginning in childhood through to adulthood, it should come as no surprise that the two share acquisition pathways.

In concluding, my position is that given the extensive knowledge base that exists with regard to antisocial and aggressive behaviours, our need for action is currently greater than our need for more research. In this paper I have attempted to demonstrate that there is substantial theoretical and empirical evidence supporting a link between human aggression and antisocial behaviour, and animal abuse. In other words, there is substantial evidence pointing to the very important role that a pattern of abusive behaviour toward animals can play in raising the alarm that other criminal behaviours are likely occurring in the same environment. In the case of children's abuse of animals, in addition to being concerned for the animals, we should be concerned for the welfare of the children as we have sufficient reason to suspect that their home environments may not be safe. In the case of older children and adolescents, we should be concerned both for the welfare of the youth themselves given the dangers they likely face in their home environments and we should be concerned for their peers since they too may be at risk in the form of bullying or related aggressive behaviours. And in the case of adults, if they are abusing animals, there is sufficient evidence to suggest that they are likely to also be engaging in other criminal behaviours, particularly human-directed aggressive behaviours. Last but not least, aggressive and abusive behaviours against animals are alarming in themselves. They are criminal behaviours that deviate from the moral and humane attitudes held by the vast majority of people worldwide and they cause unspeakable levels of suffering to our fellow sentient beings.
